# A Potential Antineoplastic Peptide of Human Prostate Cancer Cells Derived from the Lesser Spotted Dogfish (*Scyliorhinus canicula* L.)

**DOI:** 10.3390/md17100585

**Published:** 2019-10-16

**Authors:** Adrien Bosseboeuf, Amandine Baron, Elise Duval, Aude Gautier, Pascal Sourdaine, Pierrick Auvray

**Affiliations:** 1 Sciences Department, Normandy University, University of Caen Normandy (UNICAEN), Sorbonne University, French National Museum of Natural History (MNHN), University of Antilles (UA), French National Centre for Scientific Research (CNRS), French National Institute for Sustainable Development (IRD), Biology of Aquatic Organisms and Ecosystems (BOREA) Research Unit, CS14032, 14032 CAEN, CEDEX 5, France; ABosseboeuf@salud.unm.edu (A.B.); aude.gautier@unicaen.fr (A.G.); 2 Group CELLIS PHARMA, Parc Technopolitain Atalante Saint Malo, 35400 Saint Malo, France; a.baron@kelia-pharma.com (A.B.); e.duval@kelia-pharma.com (E.D.)

**Keywords:** cancer, MDA-Pca-2b, antineoplastic, marine peptide, lesser spotted dogfish (*Scyliorhinus canicula*)

## Abstract

The purpose of the present paper is to investigate the mechanism of action of a pyroglutamate-modified peptide (pE-K092D) on in vitro growth inhibition of MDA-Pca-2b prostate cancer cells. This peptide was derived from a peptide previously isolated from the testis of the lesser spotted dogfish and identified as QLTPEALADEEEMNALAAR (K092D). The effect of the peptide on cell proliferation and cell death mechanisms was studied by flow cytometry. Cellular morphology and cytoskeleton integrity of peptide-treated cells were observed by immunofluorescence microscopy. Results showed the onset of peptide induced early cytoskeleton perturbation, inhibition of autophagy, inhibition of cell proliferation and, at the end, non-apoptotic cell death mechanisms (membrane destabilization and necrosis). All those mechanisms seem to contribute to MDA-Pca-2b growth inhibition by a main cytostatic fate.

## 1. Introduction

Due to their large biodiversity, marine organisms are an interesting source of bioactive peptides exhibiting various biological characteristics, including anticancer properties [1,2,3]. Marine bioactive peptides exert different mechanisms of action on cancer cells such as plasma membrane disruption, cytoskeleton disorganization, oxidative regulation, inhibition of gene expression, cell-cycle arrest, apoptosis induction or inhibition of angiogenesis [4,5]. Among these bioactive peptides, or their derivatives, some have anti-prostate cancer properties [6]. As examples, peptides derived from Sepia ink such as the Sepia Ink Oligopeptide (SIO) and the Sepia Hydrolyzed Peptide (SHP) induced apoptosis on prostate cancer cell lines through downregulation of Bcl-2, upregulation of BAX and caspase-3 activation [7,8]. Further peptides derived from other marine mollusks have been recently reported as inducing apoptosis on prostate cancer cells [9,10].

Among marine animals, chondrichthyans seem to be an interesting class where active molecules may be found as illustrated by squalamine, an aminosterol blocking tumor-associated or age-related macular degeneration (AMD)-associated angiogenesis and also exhibiting a broad-spectrum antimicrobial activity [11,12,13,14]. Few bioactive peptides have been characterized from chondrichthyans such as the sHRSF (shark liver Hepatocyte Regeneration Stimulatory Factor) and others with angiotensin I-converting enzyme (ACE) inhibitory, antiangiogenic and anticancer activity [15,16,17,18,19,20]. Because of its abundance in European waters and the large amount of biological material available, the lesser spotted dogfish *Scyliorhinus canicula* (chondrichthyes) could be a useful marine model to further research on bioactive peptides. Previous results have shown that peptides isolated from male dogfish genital tract tissue extracts show a dose-dependent antineoplastic activity on various human cancer cell lines [21]. One of those peptides, QLTPEALADEEEMNALAAR (K092D), inhibited the in vitro proliferation of human cancer cell lines HT-29 (human colon adenocarcinoma; IC50 of 1.79 µg/µL), NCI H69 (human carcinoma, small cell lung cancer; IC25 of 1.25 µg/µL) and CCRF CEM (Human Caucasian acute lymphoblastic leukaemia; IC50 of 2.24 µg/µL). K092D also showed in vivo inhibition of HT-29-derived tumor in Nude mice model (52% of tumor volume decrease observed at day 22 after a 5-day daily 60 mg/kg peptide intravenous injection) without presenting acute toxicity (tested up to 400 mg/kg) or mutagenic effect (Ames assay) on normal cells [21].

The purpose of this work was to test whether the pyroglutamate-modified K092D peptide (pE-K092D), which is spontaneously obtained from K092D in solution (mass spectrometry analysis, data not shown), shows an efficiency on prostate cancer cells (MDA-Pca-2b cell line), prostate cancer being one of the most common cancers in men. In order to understand how pE-K092D is able to inhibit in vitro growth of the MDA-Pca-2b cell line, we first realized a kinetic study from 6 h to 96 h post-treatment to evidence the first noticeable effects. We then studied cell proliferation and cell death mechanisms by flow cytometry and cytoskeleton integrity, and cell characteristics by immunofluorescence. Finally, we investigated the cellular localization of the peptide by subcellular fractionation. Our results have shown that pE-K092D induced early cytoskeleton perturbation, inhibition of autophagy, inhibition of cell proliferation and, at the end, non-apoptotic cell death mechanisms (membrane destabilization and necrosis). All of these mechanisms seem to be contributive to the MDA-Pca-2b growth inhibition by a predominant cytostatic fate. Finally, this work proposes that dogfish tissues are of high interest in finding bioactive peptides presenting high efficiency within short treatment time.

## 2. Results

### 2.1. Decrease in Mitochondrial Activity and Cell Number Was Reported in pE-K092D-Treated Human Prostate Cancer Cells

**Figure 1:**
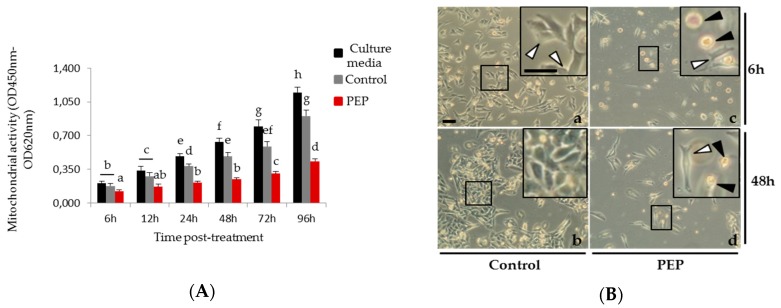
MDA-Pca-2b cells treated with pE-K092D. (**A**) Mitochondrial activity measured using the Wst-1 colorimetric assay (OD 450 nm–OD 620 nm) at 6 h, 12 h, 24 h, 48 h, 72 h and 96 h post-treatment for three different conditions of cell culture: culture media (black bars), culture media with 0.01 M ammonium bicarbonate (control, grey bars), culture media with pE-K092D at 1.6 µg/µL in 0.01 M ammonium bicarbonate (red bars). Statistical analysis were performed using the Mann and Whitney test (*p* < 0.01 between each a to h statistical group). Data were obtained from three independent experiments (N = 3; *n* = 6). (**B**) Representative phase contrast images of control cells (a, b) and pE-K092D-treated cells (c, d) at 6 h (a, c) and 48 h (b, d) post-treatment. Inserts were focused so as to distinguish adherent cell (white arrow) and round suspended cell (black arrow). Both bars represented 40 µm.

The mitochondrial potential of the cell culture was tested at 6 h, 12 h, 24 h, 48 h, 72 h and 96 h post-treatment (hpt) on cells grown with: (i) culture media, (ii) culture media and ammonium bicarbonate (0.01 M) and (iii) culture media and pE-K092D dissolved in 0.01 M ammonium bicarbonate at the final concentration corresponding to the IC50. This assay showed gradual increase of the mitochondrial activity in both controls, even if ammonium bicarbonate treatment induced a lower activity compared to culture media conditions, reflecting the cell proliferation over the considered time period. A significant decrease by half of the mitochondrial activity for pE-K092D-treated cells compared to the ammonium bicarbonate control was observed at each time, from 6 hpt (0.123 ± 0.014 for treated vs. 0.178 ± 0.022 for control) and until 96 hpt (0.432 ± 0.023 nm for treated vs. 0.904 ± 0.058 for control) (Figure 1A). Furthermore, microscopic observations at each treatment time showed that peptide-treated cells presented a decrease in cell number as well as a low rate of cellular fragments and cell death corpus, as illustrated at 6 hpt and 48 hpt (Figure 1B). Peptide-treated cells also presented more round suspended cells and less adherent cells at 6 hpt and 48 hpt, as illustrated by inserts in Figure 1B.

Those results illustrated the potential antineoplastic effect of pE-K092D on MDA-Pca-2b cells in vitro.

### 2.2. pE-K092D Induced a Generation Lag in Human Prostate Cancer Cells

**Figure 2:**
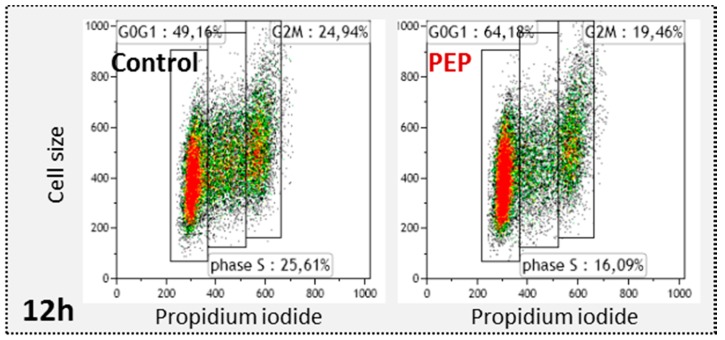
Representative flow cytometry data at 12 hpt for control and pE-K092D-treated cells (PEP).

Cell cycle repartition and corresponding cell generation were investigated on pE-K092D-treated cells and compared to control by flow cytometry at 4 h, 8 h, 12 h, 24 h, 48 h and 72 hpt (Table 1, Figure 2). At 4 hpt, peptide-treated cells included fewer in G0/G1 (60.8% for treated vs. 63.2% for control) and more cells in G2/M (24.8% for treated vs. 22.5% for control). At 8 hpt, peptide-treated cells comprised fewer cells in S (14.4% for treated vs. 16.9% for control) and more cells in G2/M (21.3% for treated vs. 18.1% for control). At 12 hpt, peptide-treated cells comprised more cells in G0/G1 (65.2% for treated vs. 50.7% for control), fewer cells in S (16.1% for treated vs. 25.6% for control) and fewer cells in G2/M (20.8% for treated vs. 23.5% for control). From 24 hpt to 72 hpt, no significant difference was observed in cell cycle repartition excepted at 72 hpt with fewer cells in S for pE-K092D-treated cells (10.6% for treated vs. 14.6% for control) (Table 1).

**Figure 3:**
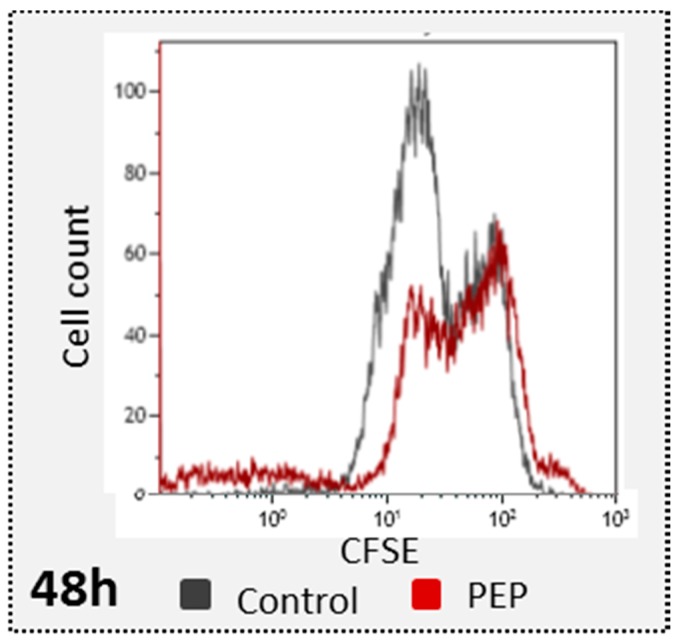
Representative flow cytometry data at 48 hpt for control and pE-K092D-treated cells (PEP).

The cell generation study showed no significant difference between control and peptide-treated cells at 6 hpt. At 12 hpt, peptide-treated cells showed more cells in generation 0 (g0) (21.6% for treated vs. 11% for control), and fewer in generations 1 (g1) (66.4% for treated vs. 74.6% for control) and 2 (g2) (9.7% for treated vs. 13.8% for control) compared to controls (Table 2). At 48 hpt, control cells progressed significantly in advanced generations compared to peptide-treated cells which developed higher percentages of cells in generations g0 (11.3% for treated vs. 1.1% for control), g1 (29.6% for treated vs. 17.1% for control) and g2 (26.5% for treated vs. 22.0% for control) and lower percentages of cells in advanced generations g3 (29.0% for treated vs. 46.5% for control) and g4 (2.2% for treated vs. 13% for control) (Table 2, Figure 3).

Those studies have shown that pE-K092D induced a durable antiproliferative effect starting at 12 hpt on MDA-PCa-2b cells.

### 2.3. pE-K092D Induced Early Autophagy Inhibition Followed by Membrane Destabilization and Necrosis

**Figure 4:**
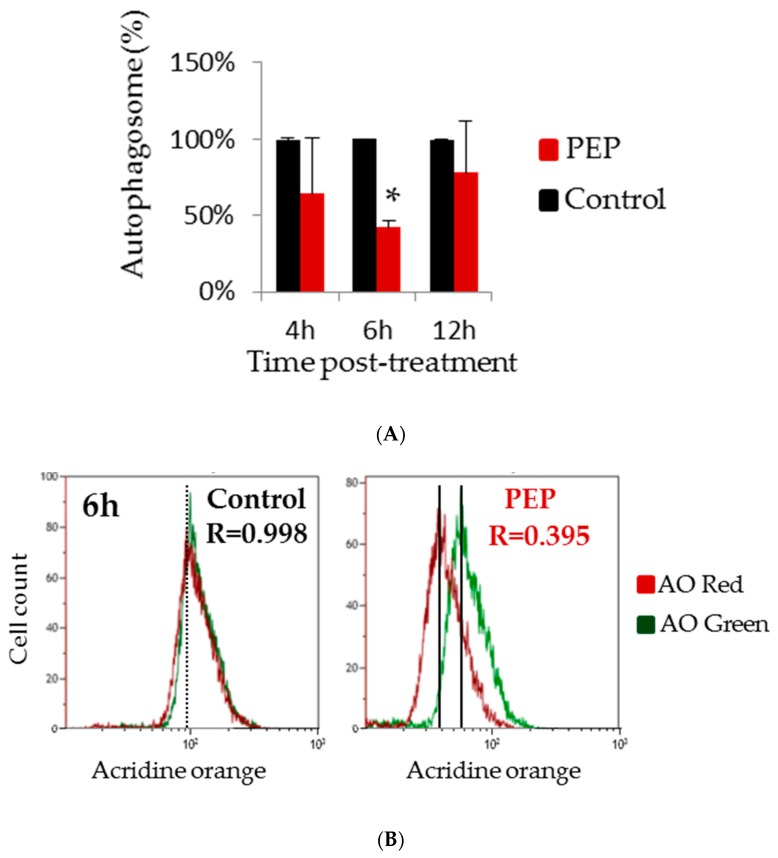
Autophagosomes analysis. (**A**) Percentages of autophagosomes in pE-K092D-treated cells (red bars) normalized to control cells (100%, black bars) as measured by flow cytometry using acridine orange assay (AO) at 4 h, 6 h and 12 h post-treatment. Statistical analysis was performed using the Mann and Whitney test (*p* < 0.01, star indicates significant differences between peptide-treated cells and control). Data was obtained from two independent experiments. (**B**) Representative flow cytometry data at 6 hpt for control and peptide-treated cells with corresponding R value of autophagosomes percentages (R = red fluorescence due to AO binding to autophagosomes / green fluorescence due to AO binding to nucleic acids).

**Figure 5:**
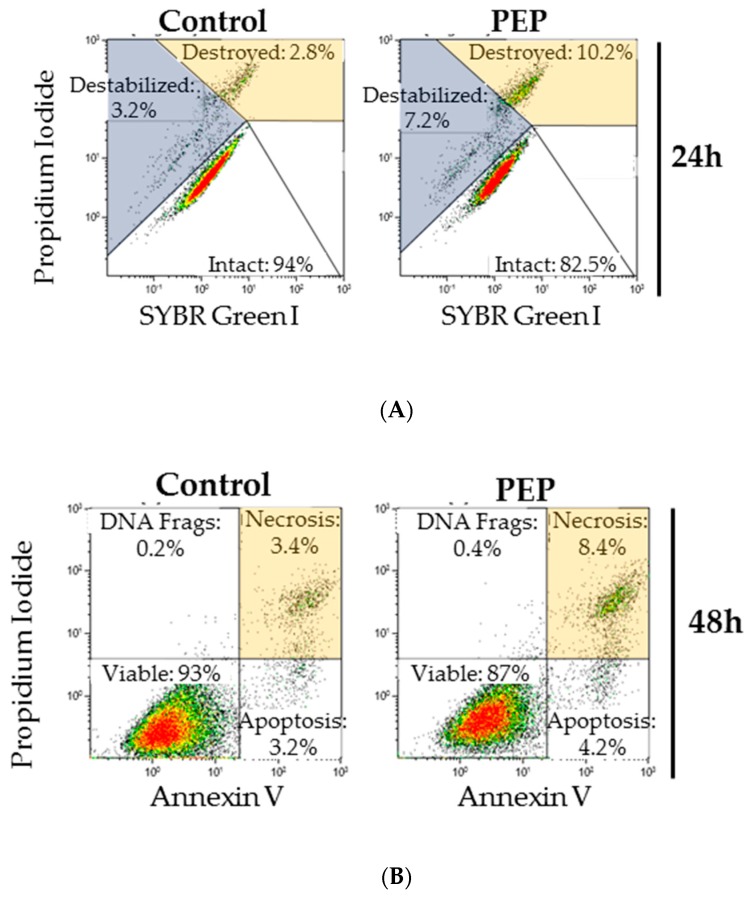
(**A**) Representative flow cytometry propidium iodide (PI)/SYBR Green I data for membrane integrity study at 24 h post-treatment illustrating the increase in destabilized and destroyed membranes in pE-K092D-treated cells. (**B**) Representative flow cytometry PI/annexin V data for cell death study at 48 h post-treatment illustrating the increase of necrosis in peptide-treated cells compared to control cells.

In order to clarify the mechanism involved in the significant antiproliferative effect of pE-K092D observed at 12 hpt, autophagy capacity of peptide-treated cells at 4, 6 and 12 hpt (Figure 4A,B) were further researched and death mechanism like apoptosis, necrosis and membrane destabilization at 24, 48 and 72 hpt (Table 3, Figure 5A,B) were investigated. Peptide-treated cells presented an early decrease of the percentage of autophagosomes observed at 6 hpt (42.3% of the control, Figure 4A), and no difference between treated and controls was observed at 4 and 12 hpt. A significant increase of destabilized membranes was observed in treated cells at 24 hpt (6.9% for treated vs. 3.0% for control), 48 hpt (7.5% for treated vs. 2.1% for control) and 72 hpt (4.8% for treated vs. 2.2% for control) (Table 3, Figure 5A). In parallel, the level of destroyed membranes also increased at 24 hpt (9.1% for treated vs. 2.2% for control), 48 hpt (3.3% for treated vs. 1.5% for control) and 72 hpt (4.6% for treated vs. 1.5% for control) (Table 3, Figure 5A). No difference between treated cells and controls was observed for the DNA fragments and for apoptosis, however a rise in necrosis levels was observed at 48 hpt (5.2% for treated vs. 2.3% for control) and 72 hpt (6.4% for treated vs. 4.0% for control) (Table 3, Figure 5B).

These results showed that pE-K092D triggered an early onset of autophagy inhibition and then induced cell death by membrane destabilization and necrosis.

### 2.4. pE-K092D Induced Early Cytoskeleton Perturbations with Particular Features

**Figure 6:**
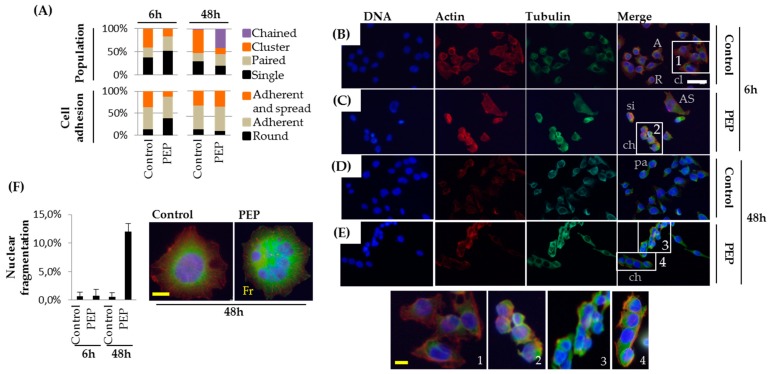
Adherence capacity, chained cells formation and nuclear fragmentation in MDA-Pca-2b cells. (**A**) Percentages of cell distribution based on their morphological characteristics in control and pE-K092D-treated cell populations (PEP) at 6 h and 48 h post-treatment. (**B**–**E**) Representative cytofluorescence with DAPI (DNA, blue), phalloidin (actin, red) and anti-tubulin antibody (tubulin, green) of control (B, D) and peptide-treated (C, E) cells at 6 hpt (B, C) and 48 hpt (D, E) are represented. si: single cell, pa: paired cells, cl—clusters of cells, ch—chained cells, R—round cells, A—adherent cells, AS—adherent and spread cells. Inserts (numbered 1–4) highlighted the difference between a cluster (1) and chained cells (2–4). The yellow scale bar represents 12.5 µm and the white bar 40 µm. (**F**) Percentages of cells with fragmented nucleus (Fr) in control and peptide-treated cell populations at 6 h and 48 h post-treatment with representative cytofluorescence. The yellow bar represents 12.5 µm.

**Figure 7:**
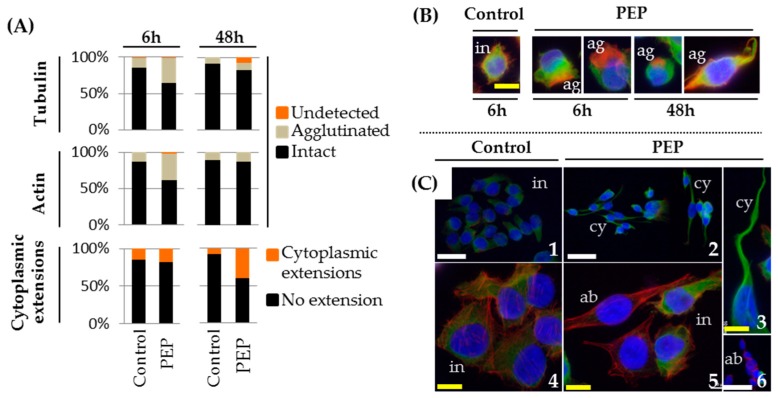
Alteration of actin and tubulin cytoskeletons and cytoplasmic extensions in MDA-Pca-2b cells. (**A**) Percentages of cell distribution based on their characteristics for microtubules, microfilaments and cytoplasmic extensions in control and pE-K092D-treated cell populations at 6 h and 48 h post-treatment. (**B**) Representative cytofluorescence with DAPI (DNA, blue), phalloidin (actin, red) and anti-tubulin antibody (tubulin, green) of control and pE-K092D-treated cells at 6 h and 48 h post-treatment showing differences between intact cytoskeleton (in) and agglutinated cytoskeleton (ag) in single isolated cells. Yellow scale bar equals 12.5 µm. (**C**) Representative cytofluorescence with DAPI (DNA, blue), phalloidin (actin, red) and anti-tubulin antibody (tubulin, green) of control (1, 4) and pE-K092D-treated cells (2–3, 5–6) at 48 h post-treatment showing differences between cells with intact cytoskeleton (in, 1, 4), cells with cytoplasmic extensions (cy, 2) and cells without detectable microtubules (ab, 5). Focus on cell with aberrant cytoplasmic extension (3) and chained cells without tubulin staining (6). The yellow bar is 12.5 µm and white bar 40 µm.

Actin filaments and microtubules as well as nuclei were observed at 6 and 48 hpt (Figure 6 and Figure 7). Comparatively to controls, at 6 hpt the pE-K092D-treated cell population presented more single cells (52% for treated vs. 38% for control) and more paired cells (31.5% for treated vs. 21.5% for control) whereas fewer clusters of cells were observed (16.5% for treated vs. 40.5% for control) (Figure 6A–C). More round cells (34.6% for treated vs. 13.3% for control) and fewer adherent and spread cells (11% for treated vs. 31.6% for control) were observed at 6 hpt in pE-K092D-treated cells than in controls (Figure 6A–C). At 48 hpt, the peptide-treated cell population was characterized by a larger number of chained cells (41.3% for treated vs. 1.8% for control, Figure 6A,D,E) and nuclear fragmentation (12.6% for treated vs. 1.4% for control, Figure 6F).

Investigation of cytoskeleton modifications by cytofluorescence observations made it possible to report agglutinations of microtubules (34.6% for treated vs. 13.3% for control) and of actin filaments (37% for treated vs. 13.3% for control) in pE-K092D-treated cells at 6 hpt (Figure 7A,B). The increase in percentage of cells presenting cytoplasmic extensions was also noticed at 48 hpt for pE-K092D-treated cells (39.5%) comparatively to control (7.3%) (Figure 7A–C). The loss of detection of microtubules in 7.8% of pE-K092D-treated cells at 48 hpt was also reported (Figure 7A–C).

**Figure 8:**
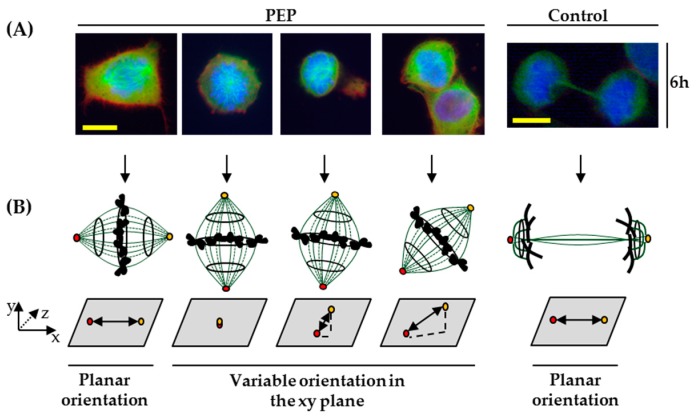
Mitotic spindle orientations in MDA-Pca-2b cells at 6 h post-treatment with pE-K092D (PEP). (**A**) Representative cytofluorescence of control and treated cells. Tubulin, actin and DNA were stained using anti-tubulin antibody (green), phalloidin (red) and DAPI (blue), respectively. (**B**) Corresponding scheme of the mitotic spindle (green bars) orientation with chromosomes (black) in the *x*–*y*–*z* axes on the culture plastic area (grey box), yellow and red circles represent the spindle poles. The yellow bar equals 12.5 µm.

Mitotic spindle orientations in the plane of the cell-culture dishes were observed at 6 hpt (Figure 8A,B). In pE-K092D-treated cells, 75% of cells in metaphase presented a perpendicularly or a variable spindle orientation in the x–y plane at 6 hpt. In contrast, only cells in telophase resulting of a planar orientation of the mitotic spindle were observed in control cells. Interestingly, subcellular fractionation (results not shown) indicated that the peptide was found to be in a five-fold higher concentration in the fraction containing nuclei and cytoskeleton than in the others at 6 hpt and 48 hpt.

These results show that pE-K092D has affected actin and tubulin cytoskeletons, cell adherence, cell morphology and the cell population characteristics with, among others, a misorientation of the mitotic spindle.

## 3. Discussion

This study reported that the first effects observed for pE-K092D treatment on MDA-Pca-2b prostate cancer cells were an inhibition of autophagy and a perturbation of the cytoskeleton including a non-planar orientation of the mitotic spindle. These effects were followed by a durable slowing-down of the cell cycle. Later on, other effects were reported such as, to a lesser extent, an increase of cell membrane destabilization and cell necrosis, cell morphology disorders and nucleus fragmentation.

Autophagy is a complex mechanism allowing organelle and protein turnovers, thereby protecting cells from various stress conditions. In cancer cells, autophagy according to its intensity can promote either cell survival allowing tumor survival and development, or cell death by apoptotic or non-apoptotic cell death (such as necrosis) [22,23,24]. This dual role of autophagy in cancer can lead to reports where its inhibition, or on the contrary its activation, is associated with a promotion of apoptosis. Thus, prostate cancer cell death through induction of autophagy, apoptosis and necrosis or disturbance of tubulin formation has been reported [25,26]. Conversely, inhibition of autophagy was also correlated with an increase of caspase-dependent apoptosis in various cells such as HeLa or lymphoma cells [27] and sometimes with necrosis in other cells such as human glioblastomes [28]. Our results have shown that autophagy inhibition was observed at 6 hpt on pE-K092D-treated cells but was not maintained and not followed by apoptosis, suggesting that this first effect was transitory. However, late necrosis was observed in pE-K092D-treated cells suggesting that the transitory autophagy inhibition may contribute to non-apoptotic cell death mechanism in MDA-Pca-2b cells.

In light of the various interactions described between the cytoskeleton and autophagy [29], the autophagy inhibition observed on pE-K092D-treated cells could be induced by cytoskeleton perturbations observed at the same time. Indeed, the concomitance between tubulin remodeling and autophagy inhibition has been reported on mouse neural cells [30]. Moreover, the cytoskeleton dynamics and the interactions between its different compounds are of course fundamental during all steps of mitosis such as for the mitotic spindle assembly, the mitotic spindle orientation or for cytokenesis [31]. As reported in many studies on various cancer cell lines, direct perturbation of the depolymerization/polymerization of microtubule induce cell cycle arrest before metaphase and are often associated with mitotic spindle aberrations [32,33,34]. Thus, the kinetics of observed effects let us hypothesize that the slowing-down of cell cycle was a consequence of the cytoskeleton perturbation. Other cell morphological characteristics similar to those we observed during our study are associated to cytoskeleton perturbation such as the chained cell formation in yeast [35] or such cytoplasmic extensions in breast cancer cells over-expressing the guanine nucleotide releasing factor (C3G) [36] or treated with the suberoylanilide hydroxamic acid (SAHA) antineoplastic drug [37]. Moreover, the subcellular fractionation experiment had shown that the peptide was mainly found in the fraction containing nuclei and cytoskeleton, which was consistent with cytoskeleton perturbation. Finally, all of our observations suggest an interaction of pE-K092D with a cytoskeleton element leading to a disruption of multiple cellular processes, including membrane destabilization that could be related to cell cortex perturbation and would finally promote the cell necrosis of some MDA-Pca-2b cells. In parallel, the strong cell cycle slowing-down observed at 12 hpt could be correlated to a cytostatic fate, which was consistent with observations showing the lack of cellular fragments and cell death corpus.

To conclude, pE-K092D is a potential antineoplastic marine peptide which inhibits the growth of MDA-Pca-2b cells in vitro by an original action mechanism with a fast and durable effect leading to various cell dysfunctions. Its main target seems to be a cytoskeleton element whose disruption would induce a strong and precocious cytostatic fate followed by a lighter cytotoxic effect. Further investigations should be performed at the molecular level to confirm these hypotheses and to identify which cytoskeleton element is involved.

## 4. Materials and Methods 

### 4.1. Cell Culture and Treatment Conditions

MDA-Pca-2b cell line was established from the bone metastasis of a 63 years old black man with androgen-independent adenocarcinoma of the prostate (cell line obtained from the global bioresource center ATCC; www.atcc.org/). This cell line expresses prostate specific antigen (PSA) and androgen receptor, grows in vitro and in vivo, and is androgen sensitive. MDA-Pca-2b cells were maintained in Ham’s F12 media (supplemented with 20% SVF, 5 mM L-Glu, 10 ng/mL EGF, hydrocortisone 100 pg/mL) at 37 °C in a 5% CO_2_ air incubator. Prior experiments, the cells were grown to about 80% confluence three times and then exposed to peptide at a concentration corresponding to IC50 (1.6 µg/µL). pE-K092D was synthetized by and purchased from Bionexus company (Strasbourg, France; 95.90% purity), stored at −20 °C and dissolved in bicarbonate ammonium (0.1 M) when needed. Results were compared with those obtained for controls, cells grown in culture media and in culture media with bicarbonate ammonium (0.1 M).

### 4.2. Mitochondrial Activity Assay

Mitochondrial activity was measured by the WST-1 colorimetric assay (Roche; Ref.: 11 644 807 001) based on the cellular capacity to metabolize the WST-1 by mitochondrial enzymatic complex. Cells were seeded into 96 well-plates (5000 cells/well) and treated in replicates for 6 h, 12 h, 24 h, 48 h, 72 h and 96 h. WST-1 was added as recommended by suppliers and plates were incubated for 2h before being analyzed in a spectrophotometer (BioRad; iMark; Marnes-la-Coquette, France). Optical density (OD) at 620 nm, corresponding to background level, was subtracted from OD at 450 nm corresponding to WST-1 degradation. Statistical analyses were performed using the Mann and Whitney test (*p* < 0.01) on three independent experiments (N = 3; *n* = 9).

### 4.3. Apoptosis/necrosis and Membranes Integrity Analysis

Both apoptosis/necrosis and membrane integrity were quantified by flow cytometry using two double stainings: propidium iodide (PI) and annexin V (AV), or PI and sybr green I (SGI) respectively. PI and SGI were both used to stain DNA, but only the SGI could pass through cellular membranes without perforation. Annexin V allowed apoptosis/necrosis detection by its high affinity to bind phosphatidylserins exposed at the extracellular side of apoptotic cells. Cells were seeded into 24 well-plates (29,688 cells/well) and treated in replicates for 6 h, 12 h, 24 h, 48 h and 72 h. Cells were then trypsinized (170 U/mL), washed and then stained in two ways: (i) apoptosis/necrosis analysis using PI and AV (both 1:500, as described by suppliers); (ii) membrane integrity analysis using SGI at 1:1 × 10^6^ and PI at 1:500 with an incubation time of 30 min in the dark. Samples were then analyzed by flow cytometry (Gallios cytometer, Beckman Coulter Life Sciences, Villepinte, France). For each sample, 20,000 events were counted and statistical analyses were performed on three independent experiments using the Mann and Whitney test (*p* < 0.05) (N = 3; *n* = 6).

### 4.4. Cell Cycle Analysis

Cell cycle repartition was investigated at 4 h, 8 h, 12 h, 24 h, 48 h and 72 h by flow cytometry with PI staining. On ethanol fixed cells, PI staining allowed DNA quantification corresponding to cell populations in G0/G1 (2C); S (between 2C and 4C) or G2/M (4C). Cells were seeded in replicates into 24 well-plates (29,688 cells/well) and treated. Then, they were trypsinized (170 U/mL), washed, fixed and stored in ethanol 70% at −20 °C. Before flow cytometry analysis, cells were washed at 37 °C and stained for 30 min in dark conditions with PI as described by suppliers. For each sample, 20,000 events were counted and statistical analysis was performed on three independent experiments using the Mann and Whitney test (*p* < 0.05) (N = 3; *n* = 6).

### 4.5. Cell Generation Study

Cell generation was determined at 6 h, 12 h and 48 h post-treatment using Carboxy Fluorescein Succinimidyl Ester assay (CFSE) with flow cytometry analysis (Gallios cytometer with Kaluza licence, Beckman Coulter Life Sciences, Villepinte, France). CFSE was sequestrated in intracellular compartment and at the cell surface during treatment time and was equally subdivided into the 2 daughter cells after mitosis. So, the first generation, corresponding to seeded cells (called g0), showed the maximum of fluorescence and for each following generation, fluorescence was divided by two. Cells were stained with 5 µM CFSE for 10 minutes at 37 °C, washed with PBS, seeded in replicates into 24 well-plates (29,688 cells/well) and treated. Cells were then trypsinized (170 U/mL), washed, and analyzed by flow cytometry. For each sample, 50,000 events were counted and statistical analyses were performed on two independent experiments using the Mann and Whitney test (*p* < 0.05) (N = 2; *n* = 4).

### 4.6. Autophagy Measurement

Autophagy, directly correlated with the quantity of autophagosomes, was investigated at 4, 6 and 12 hpt using acridine orange assay and flow cytometry analysis. Acridine orange was used to stain autophagosome organelles (red fluorescence) and nucleic acids in cytoplasm and nucleus (green fluorescence). The percentage of autophagosomes (R; R = 100% for controls) was determined by normalization of the red fluorescence by the green fluorescence: R = FL3 INT / FL1 INT. Cells were seeded in replicates into 24 well-plates (29,688 cells/well) and treated. Cells were then stained with AO (5 µg/mL) for 10 minutes at 37 °C, trypsinized (170 U/mL), washed and analyzed by flow cytometry. For each sample, 20,000 events were counted and statistical analysis was performed on two independent experiments using the Mann and Whitney test (*p* < 0.05) (N = 2; *n* = 4).

### 4.7. Immunocytochemistry Analysis

Tubulin and actin cytoskeletons were observed at 6 and 48 hpt using a mouse anti-tyrosine tubulin antibody (Sigma: T9028; L’Isle d’Abeau Chesnes, France) and phalloidin staining (5 µg/mL), respectively. Cells were seeded in replicates on microscope cover glass slides into 24 well-plates (29,688 cells/well) and treated. Cells were then fixed in 4% PFA for 10 minutes, washed in 1% BSA-PBS solution, incubated 1 h with both phalloidin (1:200) and anti-tubulin (1:200), washed, incubated with secondary antibody (1:500; goat anti-mouse IgG Alexa fluor 488; Invitrogen: A11001) for 1 h in the dark and washed. Cover glass slides were finally mounted on microscope slides with a mounting solution containing DAPI (Prolong gold antifade with DAPI; Invitrogen P36935). Observations, pictures and merge were taken with an Eclipse 80i microscope coupled to a DXM1200-C camera (Nikon, Champigny sur Marne, France). Quantification was made by taking 25 pictures per sample. Using replicates, each independent experiment comprised 50 pictures for which cells were counted using a quantification grid.

### 4.8. Subcellular Fractionation

The cellular localization of pE-K092D was investigated at 6 hpt and 48 hpt by mass spectrometry after subcellular fractionation. Cells were first collected, homogenized using a syringe with needle in a 0.25 M isotonic saccharose solution and short spin centrifuged. Pellets corresponding to undestroyed cells were isolated and supernatants were centrifuged 20 min at 1000 g and 4 °C in order to separate nuclei and cytoskeleton elements. Those pellets were then isolated and supernatants were centrifuged for another 20 min at 1,000 g and 4 °C to isolate pellets containing organelles such as mitochondria, lysosomes and peroxysomes. Supernatants were finally centrifuged for 1 h at 100,000 g and 4 °C and as a result pellets corresponded to membranes and microsomes whereas supernatants contained cytosolic fraction with few polysomes. All pellets were then suspended in a 0.1% TFA–acetonitrile solution, stored at −80 °C and analyzed by mass spectrometry.

## Figures and Tables

**Table 1:** Cell cycle repartition in G0/G1, S and G2/M (%).

	4 h		8 h		12 h		24 h		48 h		72 h	
	Control	PEP	Control	PEP	Control	PEP	Control	PEP	Control	PEP	Control	PEP
G0/G1	63.20 ± 1.41	60.78 ± 0.98 *	64.92 ± 1.71	64.24 ± 0.54	50.70 ± 2.99	65.22 ± 1.71 *	57.11 ± 1.78	55.82 ± 6.29	67.25 ± 3.49	71.10 ± 6.02	68.83 ± 3.30	72.63 ± 0.76
S	13.22 ± 1.28	13.49 ± 1.51	16.90 ± 0.91	14.36 ± 0.54 *	25.62 ± 0.13	16.06 ± 0.14 *	16.75 ± 1.23	16.77 ± 2.59	14.36 ± 1.03	11.34 ± 2.88	14.61 ± 0.85	10.62 ± 2.35 *
G2/M	22.50 ± 0.01	24.82 ± 0.53 *	18.06 ± 1.07	21.27 ± 0.02 *	23.53 ± 2.94	20.81 ± 3.48 *	26.03 ± 2.55	27.06 ± 4.07	18.28 ± 3.20	17.13 ± 3.62	16.06 ± 2.61	16.22 ± 1.87

Cell cycle repartition was measured by flow cytometry after propidium iodide (PI) staining at 4 h, 8 h, 12 h, 24 h, 48 h and 72 h post-treatment for three independent experiments on control cells and peptide-treated cells (PEP). Results in percentages were represented by mean ± SD and statistical analysis was performed using the Mann and Whitney test (* *p* < 0.05).

**Table 2:** Cell generation (%).

	6 h		12 h		48 h	
	Control	PEP	Control	PEP	Control	PEP
g0	17.5 ± 0.4	19.8 ± 1.2	11.0 ± 0.8 *	21.0 ± 1.3	1.1 ± 0.0 *	11.3 ± 2.4
g1	73.6 ± 1.6	72.7 ± 1.8	74.6 ± 2.4 *	66.4 ± 1.8	17.1 ± 1.2 *	29.6 ± 1.9
g2	8.0 ± 0.9	7.6 ± 0.4	13.8 ± 1.2 *	9.7 ± 0.9	22.0 ± 1.3 *	26.5 ± 1.4
g3	0.3 ± 0.1	0.4 ± 0.1	0.8 ± 0.1	0.6 ± 0.2	46.5 ± 2.6 *	29.0 ± 1.5
g4	0.1 ± 0.0	0.4 ± 0.1	0.1 ± 0.0	0.2 ± 0.0	13.0 ± 0.4 *	2.2 ± 0.9

Percentage of cells in four generations (g0 to g4) was measured at 6 h, 12 h and 48 h post-treatment for two independent experiments in control cells and pE-K092D-treated cells (PEP) using Carboxy Fluorescein Succinimidyl Ester (CFSE) assay and flow cytometry analysis. Results were represented by mean ± SD and statistical analysis was performed using the Mann and Whitney test (* *p* < 0.05).

**Table 3:** Membrane integrity, apoptosis and necrosis (%).

Study	Samples	24 h	48 h	72 h
Destabilized membranes	Control	3.0 ± 0.4	2.1 ± 0.4	2.2 ± 0.8
	PEP	6.9 ± 1.1 *	7.5 ± 2.3 *	4.8 ± 0.4 *
Destroyed membranes	Control	2.2 ± 0.7	1.5 ± 0.1	1.5 ± 0.4
	PEP	9.1 ± 3.7 *	3.3 ± 0.3*	4.6 ± 0.4 *
Cell Fragments	Control	3.7 ± 1.1	3.9 ± 0.2	4.0 ± 2.6
	PEP	5.1 ± 1.4	5.3 ± 2.3	5.6 ± 2.4
Apoptosis	Control	2.3 ± 0.8	1.5 ± 0.5	1.9 ± 1.1
	PEP	2.9 ± 1.3	2.8 ± 1.5	2.7 ± 0.7
Necrosis	Control	3.1 ± 0.5	2.3 ± 0.0	4.0 ± 1.2
	PEP	5.4 ± 2.6	5.2 ± 1.9 *	6.4 ± 1.1 *

Percentages of destabilized membranes, destroyed membranes, cell fragments, apoptosis and necrosis in control and pE-K092D-treated cells (PEP) at 24 h, 48 h and 72 h post-treatment. Data obtained from three independent experiments were represented by mean ± SD, and * indicates significant differences between control and peptide-treated cells (Mann and Whitney test; *p* < 0.05).
